# Rapid and Accurate Evaluation of the Quality of Commercial Organic Fertilizers Using Near Infrared Spectroscopy

**DOI:** 10.1371/journal.pone.0088279

**Published:** 2014-02-25

**Authors:** Chang Wang, Chichao Huang, Jian Qian, Jian Xiao, Huan Li, Yongli Wen, Xinhua He, Wei Ran, Qirong Shen, Guanghui Yu

**Affiliations:** 1 National Engineering Research Center for Organic-based Fertilizers, Jiangsu Collaborative Innovation Center for Solid Organic Waste Resource Utilization, Nanjing Agricultural University, Nanjing, PR China; 2 School of Plant Biology, University of Western Australia, Crawley, Australia; National Research Council of Italy, Italy

## Abstract

The composting industry has been growing rapidly in China because of a boom in the animal industry. Therefore, a rapid and accurate assessment of the quality of commercial organic fertilizers is of the utmost importance. In this study, a novel technique that combines near infrared (NIR) spectroscopy with partial least squares (PLS) analysis is developed for rapidly and accurately assessing commercial organic fertilizers quality. A total of 104 commercial organic fertilizers were collected from full-scale compost factories in Jiangsu Province, east China. In general, the NIR-PLS technique showed accurate predictions of the total organic matter, water soluble organic nitrogen, pH, and germination index; less accurate results of the moisture, total nitrogen, and electrical conductivity; and the least accurate results for water soluble organic carbon. Our results suggested the combined NIR-PLS technique could be applied as a valuable tool to rapidly and accurately assess the quality of commercial organic fertilizers.

## Introduction

**Figure 1:**
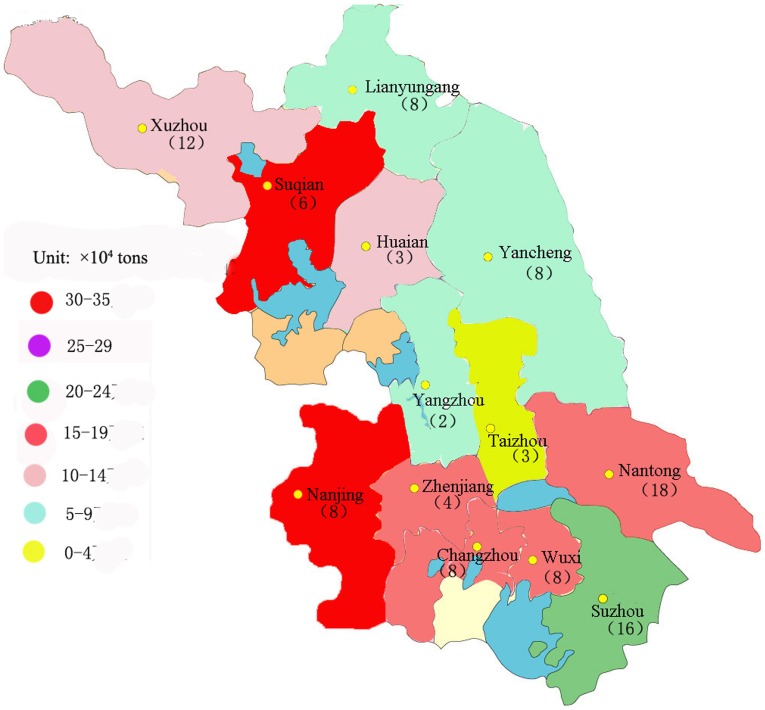
Summary of factory locations to produce commercial organic fertilizers in Jiangsu Province, China.

Composting is an inexpensive, efficient, and sustainable treatment for solid wastes [1]–[4]. The composting industry has been growing rapidly because of a boom in the animal industry in China over the past ten years [5]. Numerous large scale animal farms with more than 10,000 pigs or more than 5,000 dairy cattle have been or are being established in east China, resulting in a large amount of animal manure, which is a major pollutant if not treated and utilized as a fertilizer [5]. Each year, more than 100 factories produce over 5 thousand tons of commercial organic fertilizers in Jiangsu Province, China. Consequently, the total amount of commercial organic fertilizers produced by subsidized composting facilities is more than 2 million tons per year over there (Fig. 1). The difference between commercial organic fertilizer and compost is that the former is referred to compost products entered into the market and having a trademark on the package, while the latter is referred to materials produced during the composting process.

Nevertheless, the application of immature compost can result in inhibited seed germination, root destruction, suppressed plant growth, and a decrease in the oxygen concentration and redox potential [5]–[7]. The main difference between composts and commercial organic fertilizers is the complexity of the raw materials of the latter, and no studies have investigated if such inhibitions apply to the commercial organic fertilizers that are currently used in China. Therefore, assessing the quality of commercial organic fertilizers is of the utmost importance for achieving high quality marketable fertilizers.

Various parameters, such as the moisture, total organic matter (TOM) content, pH, water soluble organic carbon (WSOC), water soluble organic nitrogen (WSON), pH, electrical conductivity (EC), and germination index (GI), are commonly used to evaluate the compost quality [1], [3]–[5], [8]. However, all these approaches are time-consuming or expensive when a large number of samples are involved [3], [4], [9]. Near infrared (NIR) spectroscopy has many advantages over traditional chemical analyses, such as its ease of sample preparation, rapid spectrum acquisition, non-destructive nature of the analysis, and the portability of the technology [9]. A number of investigators have shown that NIRS (near infrared reflectance spectroscopy) can be applied to rapidly assess compost quality during composting [10]–[18]. However, it is unclear whether NIRS can also be used to rapid assess the quality indices of commercial organic fertilizers.

The objectives of this study were therefore to search the feasibility of rapidly assessing the essential quality indices of commercial organic fertilizers produced from different raw materials using NIR. For this purpose, a total of 104 commercial organic fertilizers were collected from full-scale compost factories, which are distributed in 13 regions in Jiangsu Province, China (Fig. 1). The measured chemical and biological parameters include the moisture content, TOM, TN, WSOC, WSON, pH, EC, and GI. A combination of NIR spectra with partial least squares (PLS) analysis was applied to rapidly evaluate the quality of commercial organic fertilizers.

## Materials and Methods

### Sample collection and pretreatment

A total of 104 commercial organic fertilizers were collected from full-scale compost factories (Fig. 1). These factories treat organic matter from animal (chicken, cattle, duck, pig, etc.) manure and other agricultural organic (straw from wheat, corn, rice, etc.) residues. No specific field permits were required for this study. The land accessed is not privately owned or protected. These factories produce approximately 0.5 to 1.5 million tons of commercial organic fertilizers per year.

All organic fertilizer products were identified and collected during factory packaging after being screened and crashed. The collected products, as granular and powered fertilizers that were thus more uniform than those during composting (see Figure S1), were then transported to the lab, air-dried, and stored at 4°C for further analysis. Meanwhile, the samples were mixed and divided into four equal subsamples by the quartile method. The subsamples were then ground (0.15 mm sieve) for the determination of their chemical and biological indices and the near infrared spectra.

### Chemical and biological indices analysis

All chemical analyses were conducted in duplicate using analytical grade chemicals. The TOM in these commercial organic fertilizers was determined by the loss on ignition at 550°C for 4 h, according to [3]. The TN was analyzed using a Perkin-Elmer 2400 CHN elemental analyzer [3], [4]. The moisture was measured by oven drying at 105°C to a constant weight. The EC and pH were measured in a 1∶10 (w/v) water extract [5]. For the WSOC and WSON analysis, 5 g samples were shaken with 50 ml of deionized water (1∶10 w∶v) for 2 h, the resulting extracts were centrifuged at 3,500 rpm for 30 min, filtered through 0.45 µm membranes and then determined using a TOC/TN analyzer (multi N/C 3000, Analytik Jena AG, Germany) [3], [4], [5]. The GI measurement was with *Lepidium sativum* L. seeds [3].

### Spectroscopic measurement

The spectra of NIR were recorded at 1.4 nm intervals from 350 to 1,000 nm and 2 nm intervals from 1,000 to 2,500 nm using a FieldSpec^@^ 3 NIR spectrometer (ASD Inc, USA). Approximately 30 g of dried sample were scanned in a 6 cm diameter sample cell with a quartz window at 18–22°C. A dark reference measurement was conducted for noise and ambient temperature correction every 30 min. Background correction was performed as a total of 64 scans which were averaged before each sample being scanned. After using the ViewSpecPro software (ASD Inc, USA), each sample was remixed, rescanned five times, and then averaged. The recorded spectral data were processed and stored as the reflectance (R) and then converted to the absorbance A (A = log 1/R).

### Partial least squares regression (PLS) analysis

The PLS regressions were used to construct the model between the laboratory parameters and NIR spectral data. A cross-validation was performed to select the optimal number of terms in the equation and to avoid over-fitting. The data pre-processing and model development was conducted using the spectroscopic software Unscrambler Trial 9.7 (CAMO Inc, Norway). A validation set, composed of independent samples, was applied to estimate the prediction accuracy of the calibration models. In this study, all 104 samples were randomly divided into a calibration set (78 samples) and a validation set (26 samples). This method had been applied by several publications [19]–[21]. To restrain invariable background signals and to improve the visual resolution, the second derivative spectra of each sample were used for further calibration and validation. The selection of the models developed was based on the values of the coefficient of determination for the calibration set (*R* ^2^) and the root mean square error in the cross validation (RMSECV), the determination coefficient of the validation (*r* ^2^) and the RPD (ratio of standard error of performance to standard deviation). The RPD is the ratio of the standard deviation (SD) in the validation set over the root mean squared error of the prediction (RMSEP). The bias and the slope value were used to evaluate the usefulness of NIRS for determining the selected quality indicators of commercial organic fertilizers.

The formula for the root mean standard error of calibration (RMSEC) is:
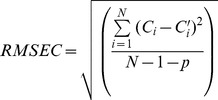
(1)where *C*_i_ is the known value, *C*′_i_ is the value calculated using the calibration equation, *N* is the number of samples, and *p* is the number of independent variables in the regression.

The root mean standard error of prediction (RMSEP) estimates the prediction performance during the validation step of the calibration equation:
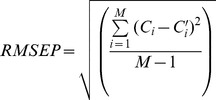
(2)where *M* is the number of samples in the prediction set.

## Results and Discussion

### Chemical and biological indices

The minimum, maximum, mean, and standard deviation (SD) of the quality indices of commercial organic fertilizers in the calibration sets and validation sets are shown in Table 1. This data set represented a wide range of compositions. For all the 104 sampled fertilizers, the mean value and SD of the quality indices were 3.95±1.70% of moisture, 357.63±76.90 g TOM/kg, 19.52±16.99 g TN/kg, 4.87±9.54 g WSOC/kg, 2.48±5.09 g WSN/kg, 6.40±0.92 pH, 5.56±2.55 mS/cm of EC, and 61.30±20.03% of GI.

**Table 1:** Composition statistics for quality indices in commercial organic fertilizers.

Parameters	Calibration set (*n* = 78)					Validation set (*n* = 26)			
	Outliers	Min	Max	Mean	SD	Min	Max	Mean	SD
Moisture (%)	2	2.43	7.34	3.73	0.99	2.57	6.64	3.84	0.82
TOM (g/kg)	2	280.82	534.54	347.86	51.18	288.30	498.35	353.82	42.30
TN (g/kg)	0	4.30	128.56	19.89	19.07	5.74	46.36	18.41	8.18
WSOC (g/kg)	7	0.11	28.34	3.75	5.66	0.17	17.47	3.80	4.58
WSON (g/kg)	10	0.08	13.97	1.95	2.79	0.18	8.44	1.89	2.35
pH	2	5.09	9.90	6.36	0.97	5.23	9.22	6.48	0.80
EC (mS/cm)	0	3.27	21.91	5.61	2.86	3.49	9.58	5.39	1.23
GI (%)	0	33.98	137.96	61.18	21.18	35.95	117.99	61.66	16.48

Abbreviations: EC, electrical conductivity; GI, germination index; SD, standard deviation, TN, total nitrogen; TOM, total organic matter content; WSOC, water soluble organic carbon; WSON, water soluble organic nitrogen.

This wide variability in the quality indices of the commercial organic fertilizers allowed us to successfully build a correlation between the NIR spectra and the compost quality indices [3]. Samples with a difference between the reference and predicted values were considered outliers and thus excluded during the calibration process. The removal of outliers was on the basis of being labeled as compositional outliers based on the criterion that if the predicted versus actual difference for a sample was 3 SD or more from the mean difference [13]. For the quality indices, two outliers were removed for the moisture, TOM, and pH; 0 for TN, EC, and GI; 7 for WSOC; and 10 for WSON (Table 1).

### NIR spectra

**Figure 2:**
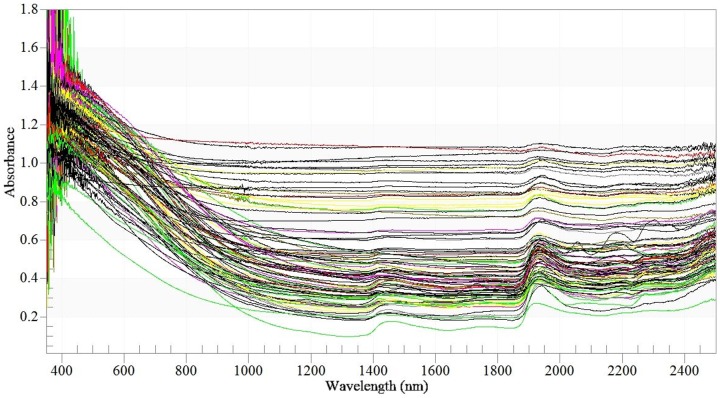
Spectra of NIR of a total 104 commercial organic fertilizers.

All the NIR spectra of the collected commercial organic fertilizers could be divided into two groups of signal with different slopes under 1,400 nm, i.e., one group presented an increased curvature and the another one was more flat. Meanwhile, the former had a significant absorbance peak at wavelengths of approximately ∼1,420 nm, but while the latter had only a small absorption at this position. The second significant spectral peak was at approximately ∼1,950 nm (Fig. 2). The absorbance band at 1,420 nm is usually assigned to the O–H and aliphatic C–H, while the band at 1,950 nm is associated with the amide N–H and O–H [20], [22], [23]. Note that the absorption peaks were heavily overlapped, mainly because the near-infrared spectrum contains all strength information of the chemical bond, chemical composition, electronegativity, etc. Meanwhile, other interference information, such as scattering, diffusion, special reflection, surface gloss, refractive index, and reflected light polarization, affects the near-infrared spectrum [24], [25]. Thus, the quantitative predictions are difficult directly through NIR spectra alone. Multivariate analyses are required to discern the response of properties of commercial organic fertilizers from spectral characteristics with the support of chemometric methods, e.g., PLS analysis.

### Partial least square calibration and validation

Based on the guideline proposed by Saeys et al. [26], the accuracy of the predictions for the calibration model is classified as excellent when *r* ^2^>0.90, good when 0.81<*r* ^2^<0.90, moderately successful when 0.66<*r* ^2^<0.80, and unsuccessful when 0.50<*r* ^2^<0.65. Meanwhile, according to Albrecht [9] and Chang et al. [27], the accuracy of the PLS model and prediction was considered good for RPD >2, acceptable for 1.4< RPD <2, and unreliable for RPD <1.4.

**Figure 3:**
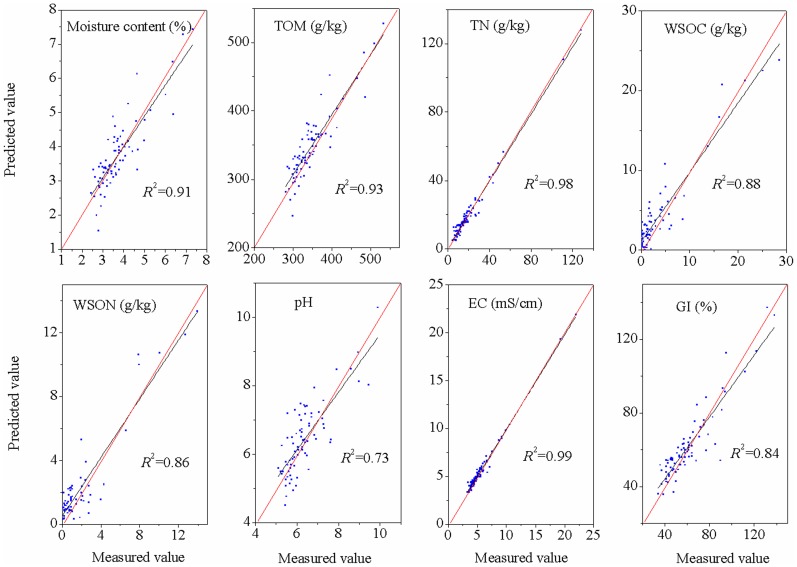
Relationships between the measured and predicted values of the quality indices of commercial organic fertilizers for the calibration data set. The red line represents the best fit. Abbreviations: EC, electrical conductivity; GI, germination index; SD, standard deviation, TN, total nitrogen; TOM, total organic matter content; WSOC, water soluble organic carbon; WSON, water soluble organic nitrogen

**Figure 4:**
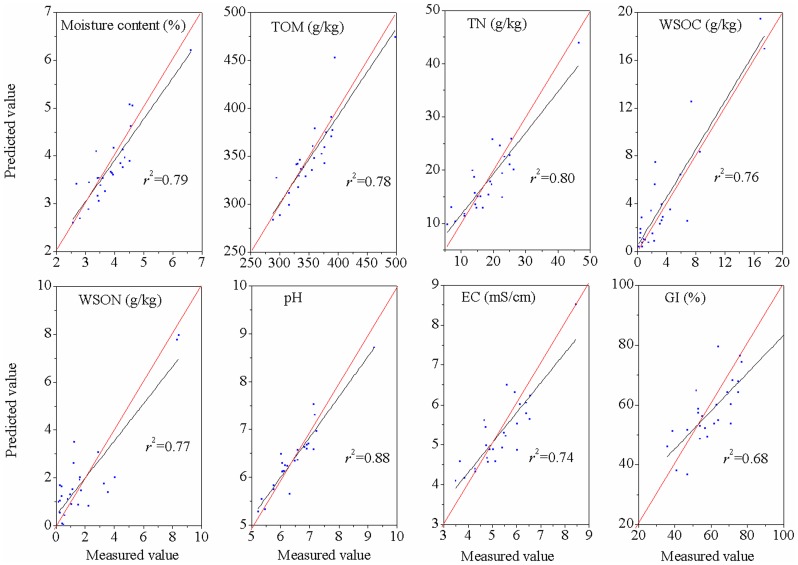
Relationships between the measured and predicted values of the quality indices of commercial organic fertilizers for the prediction data set. The red line represents the best fit. Abbreviations: TOM, total organic matter content; TN, total nitrogen; WSOC, water soluble organic carbon; WSN, water soluble nitrogen; EC, electrical conductivity; GI, germination index.

In this study, the results of the NIRS calibration and validation for the quality indices of commercial organic fertilizers are listed in Table 2 and Figures 3–4. The NIR calibrations allowed accurate predictions of the TOM, WSON, pH, and GI (*R* ^2^ = 0.73–0.93 and RPD  = 1.47–2.96). The results were less accurate for the moisture (*R* ^2^ = 0.91, *r* ^2^ = 0.79, RPD  = 2.22), TN (*R* ^2^ = 0.98, *r* ^2^ = 0.80, RPD  = 2.25) and EC (*R* ^2^ = 0.99, *r* ^2^ = 0.74, RPD  = 2.27). However, the WSOC had the worst prediction, with *R* ^2^ = 0.88, *r* ^2^ = 0.76 and RPD  = 2.10. Therefore, predictions were moderately successful for the moisture, TOM, TN, WSON, pH, EC, and GI, but unsuccessful for WSOC.

**Table 2:** NIRS calibration and validation results for quality indices of commercial organic fertilizers.

Parameters	Calibration set			Validation set				
	PC	*R* ^2^	RMSECV	*r* ^2^	RMSEP	RPD	Bias	Slope
Moisture content (%)	8	0.91	0.58	0.79	0.37	2.22	−0.07	0.86
TOM (g/kg)	8	0.93	22.92	0.78	19.38	2.18	−3.60	0.91
TN (g/kg)	17	0.98	3.08	0.80	3.63	2.25	−0.29	0.77
WSOC (g/kg)	10	0.88	3.64	0.76	2.18	2.10	0.24	1.04
WSON (g/kg)	8	0.86	1.81	0.77	1.60	1.47	0.05	0.74
pH	4	0.73	0.96	0.88	0.27	2.96	−0.07	0.84
EC (mS/cm)	17	0.99	0.46	0.74	0.54	2.27	−0.04	0.78
GI (%)	18	0.84	4.81	0.68	9.52	1.73	−2.68	0.63

Note that: TOM, total organic matter; TN, total nitrogen; WSOC, water soluble organic carbon; WSON, water soluble organic nitrogen; EC, electrical conductivity; GI, germination index; PC, number of principal component; *R* ^2^, the coefficient of determination for the calibration set; RMSECV, the root mean square error in cross validation; *r* ^2^, the coefficient of determination for the validation set; RMSEP, room mean squared error of prediction; RPD, the ratio of the standard deviation in the validation set over the room mean squared error of prediction. The calibration and validation of some indices (i.e., moisture content, TOM, WSOC, pH) was conducted using the second derivative with SNV, but that for the others was conducted using only the second derivative.

Previous studies have demonstrated that the NIR-PLS was successful in predicting some parameters such as nitrogen (N), carbon (C), C/N, humic acid, pH, respiration, and composting time during the composting process. For example, Saeys et al. [26] developed calibrations using the PCA and PLS regressions for the moisture (*r* ^2^ = 0.91, RPD  = 3.22), TOM (*r* ^2^ = 0.90, RPD  = 3.00) and TN (*r* ^2^ = 0.86, RPD  = 2.63) in pig manure using a mobile spectroscopy instrument. Huang et al. [28] obtained calibrations for the moisture (*r* ^2^ = 0.98, RPD  = 7.48), pH (*r* ^2^ = 0.62, RPD  = 1.63), EC (*r* ^2^ = 0.90, RPD  = 3.10), and TN (*r* ^2^ = 0.97, RPD  = 6.11) in animal manure (cattle, chicken, and pig manures) composts using the NIR-PLS method. Vergnoux et al. [18] obtained excellent calibrations using PCA and PLS regressions in sewage sludge compost for the moisture content (*r* ^2^ = 0.91), TN (*r* ^2^ = 0.98), and pH (*r* ^2^ = 0.92). Albrecht et al. [9] evaluated the biological and chemical changes during the composting process of green waste and sewage sludge using NIR and found that the NIR calibrations successfully allowed accurate predictions of N, C, the C/N ratio, humic acid (HA), pH, and composting time, but were less accurate for the OM, protease, acid, and alkaline phosphatase and unsatisfactory for fulvic acid. Soriano-Disla et al. [29] obtained moderately successful predictions for WSOC in compost (*r* ^2^ = 0.75, RPD  = 1.70) and in sewage sludge (*r* ^2^ = 0.60, RPD  = 1.60). However, these investigations were conducted in samples during composting, and no report has applied the NIR-PLS to predict the indices of commercial organic fertilizers.

An obvious difference between samples from the whole composting process and those from commercial organic fertilizers is the wide variability of the ingredients used in the elaboration of the composting heaps. Therefore, obtaining good correlations was more difficult for commercial organic fertilizers used in this study. The schematic of rapidly evaluating the quality of commercial organic fertilizers using near infrared spectrometer was given in Figure S2. The results in this study indicated for the first time that the indices of commercial organic fertilizers could be well-evaluated by the NIR with PLS regression method.

## Conclusions

In this study the NIR spectroscopy combined with PLS analysis has been developed as an alternative method to traditional chemical analysis for rapidly and accurately predicting the essential quality indices of commercial organic fertilizers. In general, the NIR-PLS technique provided accurate predictions of the TOM, WSON, pH, and GI; less accurate results for the moisture, TN, and EC; and the worst results for WSOC. As a result, we suggest the NIR spectroscopy with PLS analysis may be used as a valuable industrial and research tool to rapidly and accurately assess the quality of commercial organic fertilizers.

## Supporting Information

Figure S1 **Typical commercial organic fertilizers, including powered (A, C) and granular (B, D) fertilizers.** These photos suggest that the commercial organic fertilizers are more evenly than samples from the composting process.
(TIF)

Figure S2 **Schematic of rapid evaluating the quality of commercial organic fertilizers using near infrared spectrometer.**
(TIF)
