# Supplementary figures and images for: Rapid and Accurate Evaluation of the Quality of Commercial Organic Fertilizers Using Near Infrared Spectroscopy
Source: PLoS One. 2014 Feb 25;9(2):e88279. doi: 10.1371/journal.pone.0088279 (PMC3934863; doi:10.1371/journal.pone.0088279)

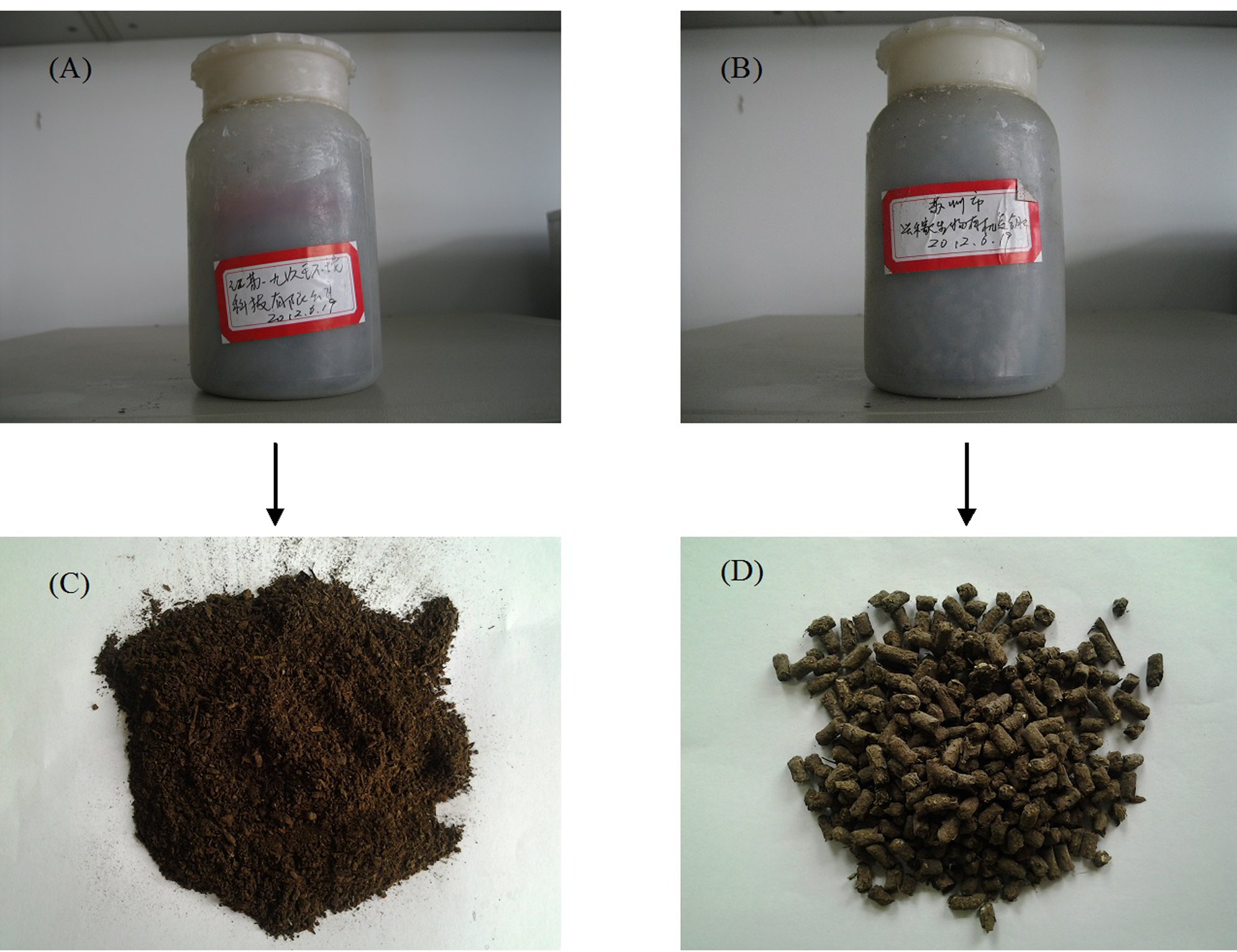

Supplement: Figure S1 — Typical commercial organic fertilizers, including powered (A, C) and granular (B, D) fertilizers. These photos suggest that the commercial organic fertilizers are more evenly than samples from the composting process. (TIF) [file pone.0088279.s001.tif]

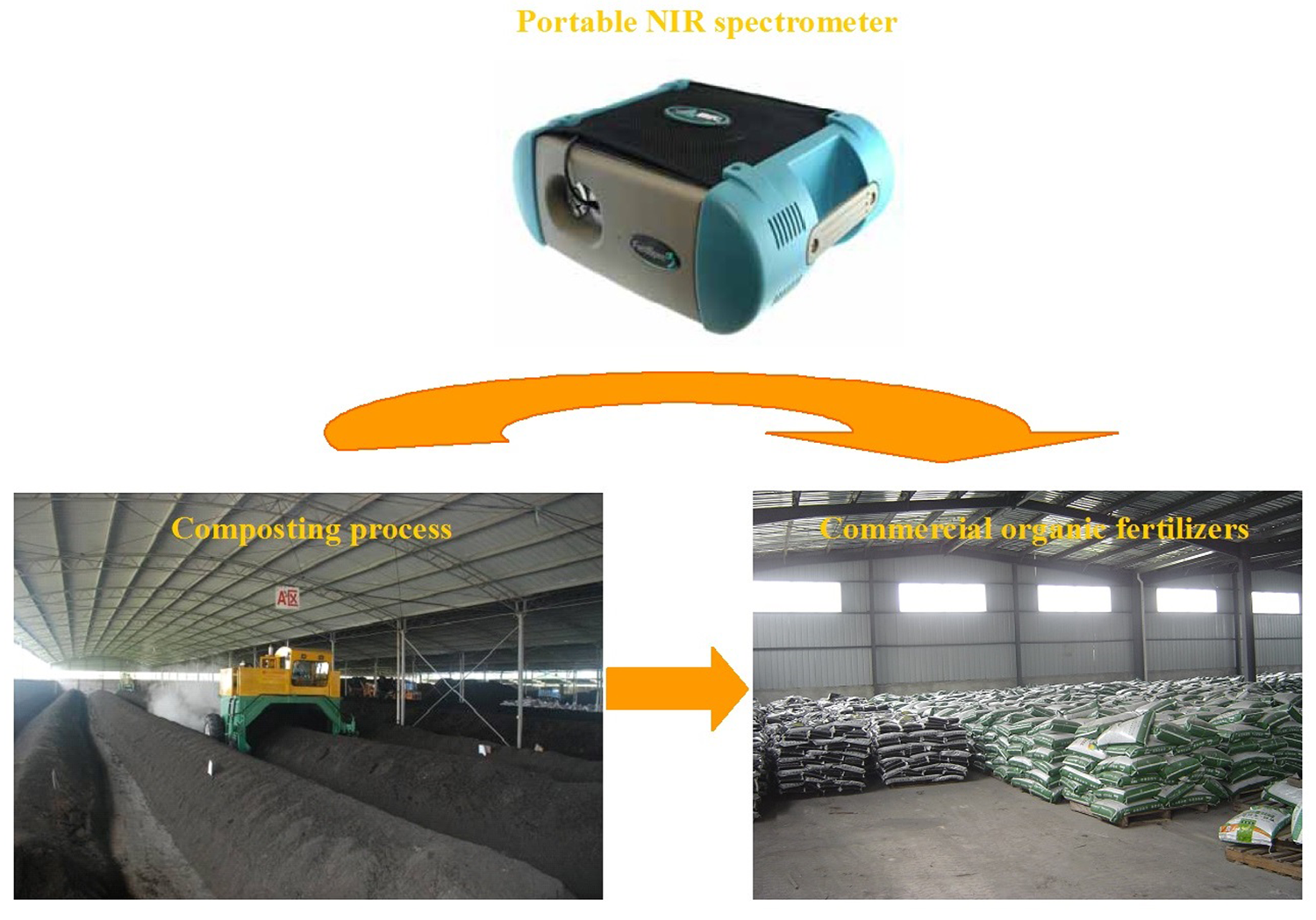

Supplement: Figure S2 — Schematic of rapid evaluating the quality of commercial organic fertilizers using near infrared spectrometer. (TIF) [file pone.0088279.s002.tif]
